# Interpretable Transfer Learning for Cancer Drug Resistance: Candidate Target Identification

**DOI:** 10.3390/cimb47090753

**Published:** 2025-09-12

**Authors:** Wenjie Zhang, Xisong Wu, Liang Chen, Xinyue Wan

**Affiliations:** 1 School of Medicine, Chongqing University, Chongqing 400030, China; 202337021070t@stu.cqu.edu.cn; 2 College of Life Science, Chongqing Normal University, Chongqing 401331, China; 2024110513053@stu.cqnu.edu.cn

**Keywords:** deep learning, gene expression profile, drug resistance, predictive markers, model interpretation

## Abstract

Tumor drug resistance exhibits substantial heterogeneity across cancer types, reflecting distinct molecular mechanisms in each malignancy. To characterize this complexity, we developed a pan-cancer transfer learning framework that integrates bulk RNA-seq data with a residual variational autoencoder (Res VAE) backbone. Five models were trained on the Genomics of Drug Sensitivity in Cancer (GDSC) dataset, which includes drug response profiles for 72 chemotherapeutic agents. Among them, three models are specially designed by incorporating variational autoencoders and large pretrained models (LLMs): the LLM large VAE (VAE_LL), the LLM small VAE (VAE_LS), and the LLM distillation VAE (VAE_LD). Random Forest (RF) and eXtreme Gradient Boosting (XGB) were included as ensemble learning baselines. After internal cross-validation, the top four models (VAE_LL, VAE_LD, XGB, and RF) were applied to five representative TCGA cohorts comprising 1,836 patients. For each cancer type, resistance to nine clinically relevant first-line drugs was modeled, resulting in 180 drug–cancer prediction tasks. Among all models, VAE_LD achieved the best overall performance, with a mean AUC of 0.81 and an F1 score of 0.92 on the GDSC benchmark, and maintained strong predictive power in the clinical validation phase. Interpretation analyses identified tumor-specific resistance biomarkers with clinical significance. In lung adenocarcinoma, elevated expression of *TFF1* was repeatedly associated with resistance to Gefitinib and correlated with poor patient prognosis, indicating its potential as a therapeutic target. In glioblastoma, *OPALIN*, *LTF*, *IL2RA*, and *SLC17A7* were implicated in Temozolomide resistance through pathways related to epithelial differentiation and angiogenesis. In conclusion, the VAE_LD model offers a high-performing and interpretable approach for predicting drug resistance across multiple tumor types. It supports the identification of clinically actionable biomarkers and provides a robust framework for precision oncology applications.

## 1. Introduction

Tumor drug resistance remains one of the most formidable obstacles in cancer therapy. It markedly reduces treatment efficacy and severely compromises patients’ long-term prognosis and quality of life [1]. In recent years, the tumor immune microenvironment (TIME) has emerged as a major contributor to therapeutic resistance, with immune evasion and immunosuppressive conditions playing pivotal roles in tumor progression. In addition, a variety of biological processes (including epigenetic dysregulation, extracellular vesicle-mediated drug efflux, and malfunction of membrane transporters) have been identified as key drivers of resistance across multiple malignancies [2,3]. These interconnected mechanisms contribute to the significant heterogeneity observed in treatment responses and present major challenges for traditional predictive models.

Conventional biomarker-based approaches often rely on single-gene indicators, which are insufficient to capture the complex, multifactorial nature of drug resistance phenotypes. In contrast, the advent of high-throughput transcriptomic technologies, particularly bulk and single-cell RNA sequencing, coupled with artificial intelligence (AI) methodologies has enabled the development of models capable of capturing high-dimensional gene expression patterns to more comprehensively predict therapeutic resistance [4].

Recent advances in foundation models, such as scFoundation and Geneformer, have demonstrated strong capabilities in extracting biologically meaningful transcriptional embeddings from single-cell data [5]. Building upon these representations, deep learning architectures such as variational autoencoders (VAEs) [6], as well as ensemble machine learning algorithms including Random Forest (RF) and eXtreme Gradient Boosting (XGB) [7], have shown promise in integrating bulk RNA-seq data for drug sensitivity prediction.

However, most prior studies have primarily focused on transferring transcriptomic features between bulk and single-cell datasets without incorporating real-world clinical outcomes [8]. In clinical practice, patient-level variables (such as first-line therapeutic regimens, treatment responses, and survival outcomes) are essential for model validation and translational relevance. These variables, however, remain underutilized in many current computational approaches [9]. Moreover, few AI-based frameworks directly combine drug resistance prediction with prognosis modeling at the patient level.

To address these limitations, we developed three novel deep learning models based on scATD and benchmarked them against two widely used classical methods. The proposed models include the following: (i) the LLM-large VAE (VAE_LL), a variational autoencoder constructed using scFoundation-derived transcriptomic features; (ii) the LLM-small VAE VAE_LS), which leverages Geneformer embeddings; (iii) the LLM-distillation VAE (VAE_LD), a residual-structured autoencoder optimized through a knowledge distillation strategy. In addition, RF and XGB were used as baseline ensemble learning models. All five models were trained using the Genomics of Drug Sensitivity in Cancer (GDSC) dataset, which encompasses 72 chemotherapeutic agents and represents a diverse range of tumor types and resistance profiles. Based on cross-validated performance metrics, the four best-performing models (VAE_LL, VAE_LD, RF, and XGB) were selected for subsequent clinical validation [10].

For external evaluation, these four models were applied to bulk RNA-seq and clinical survival data from five representative cancer types in The Cancer Genome Atlas (TCGA), namely lung adenocarcinoma (LUAD, *n* = 589), glioblastoma (GBM, *n* = 175), acute myeloid leukemia (LAML, *n* = 151), melanoma (SKCM, *n* = 473), and stomach adenocarcinoma (STAD, *n* = 448), resulting in a total cohort of 1836 patients. For each cancer type, resistance to nine clinically relevant first-line drugs (Cediranib, Dabrafenib, Dinaciclib, Entinostat, Foretinib, Gefitinib, Temozolomide, Trametinib, and AZD2014) was modeled, yielding a total of 180 drug–cancer prediction tasks [11]. These drugs span multiple therapeutic categories, including anti-angiogenic agents, cell cycle inhibitors, epigenetic regulators, DNA damage response modulators, and inhibitors of key oncogenic signaling pathways, thereby providing a comprehensive platform for biomarker discovery and resistance stratification.

Given the complexity of gene–gene interactions in drug response, we further incorporated explainable AI (XAI) methodologies, including Integrated Gradients, GradientSHAP, and TreeSHAP, to interrogate the molecular mechanisms underlying each model’s predictions [12,13]. The identification of key features was supported by gene importance ranking, interaction network construction, and pathway enrichment analysis, which together reinforced the biological plausibility of the identified biomarkers.

Across various tumor–drug contexts, the models demonstrated robust predictive accuracy and reproducible biomarker identification. In LUAD, for example, elevated expression of *SFTPC*, a marker of the terminal respiratory unit (TRU) subtype, was associated with resistance to Cediranib [14]. Although *SFTPC* is generally considered a favorable prognostic indicator, our results suggest that its high expression may confer intrinsic resistance by promoting vascular normalization and reducing dependence on VEGF-A signaling, a key target of Cediranib, which primarily inhibits VEGFR-1, -2, and -3 [15]. Dysregulated VEGF–VEGFR signaling is a canonical driver of therapeutic resistance at the interface of tumor vasculature and immunity. Excess VEGF fosters structurally and functionally abnormal vessels that aggravate hypoxia, hinder the intratumoral delivery of cytotoxic and targeted agents, and reinforce immune escape by limiting effector cell trafficking and antigen presentation. Conceptually, restoring vessel structure and function through vascular normalization can reopen a therapeutic window in which perfusion and oxygenation are improved, thereby potentiating the activity of chemotherapy, radiation therapy, and immune checkpoint blockade [16]. At the same time, resistance to anti-angiogenic strategies frequently emerges and may be shaped by isoform-level heterogeneity within the VEGF family, exemplified by VEGF165b, an alternatively spliced, anti-angiogenic variant that competes for VEGFR binding and can attenuate pro-angiogenic signaling. These considerations underscore the need to model VEGF pathway dependence when predicting drug resistance and to interpret anti-angiogenic contexts alongside immune and stromal features [17]. Prognostic biomarkers do not necessarily translate into predictive markers for treatment response, and therapeutic pathway dependencies must be accounted for when designing individualized treatment strategies.

In summary, the VAE_LD architecture proposed in this study demonstrated the best overall performance, achieving accurate prediction of multidrug resistance across both cell line and patient-derived datasets. Beyond predictive accuracy, this study provides a unified and interpretable pipeline that enables systematic identification of resistance-associated biomarkers and facilitates the elucidation of their underlying biological mechanisms.

## 2. Materials and Methods

### 2.1. Datasets

The bulk RNA-seq data used for pre-training in this study were obtained by integrating the shared gene sets from the Genomics of Drug Sensitivity in Cancer (GDSC) and the Cancer Cell Line Encyclopedia (CCLE) databases. The GDSC dataset was employed to perform 5-fold cross-validation for training drug sensitivity prediction models, involving a total of 1280 cancer cell lines and 72 anticancer drugs. For external validation, bulk RNA-seq data were retrieved from The Cancer Genome Atlas (TCGA) database (https://portal.gdc.cancer.gov/, 15 February 2025), covering five distinct cancer types with diverse tissue origins and molecular profiles: non-small cell lung cancer, glioma, melanoma, gastric cancer, and leukemia. Corresponding clinical information such as overall survival, treatment records, and other metadata was also collected. The TCGA bulk transcriptomic data were used as an independent validation set to assess the model’s predictive performance. Furthermore, the clinical variables and model-identified key genes or pathways were incorporated into survival analysis, aiming to provide additional support for the biological interpretability and clinical relevance of the proposed framework.

### 2.2. Model Architecture

We adopted and retrained the architectures of scATD (previously reported by Zhou et al.) to construct VAE_LL, VAE_LS, and VAE_LD in fitting bulk RNA-seq data [10]. The core of these models is based on the Res-VAE architecture, originally proposed by Luo et al., which was designed to perform dimensionality reduction for high-dimensional biological data [18].

To evaluate model interpretability, we employed two widely used algorithms: Integrated Gradients (IG) and GradientSHAP. Additionally, we incorporated two representative classical machine learning models, Random Forest (RF) and eXtreme Gradient Boosting (XGB), along with their respective interpretability tools: TreeSHAP for RF and the built-in explainability module of XGB.

By comparing the predictive performance and interpretability of these five commonly used model architectures in the context of drug resistance prediction, we aimed to identify the most effective modeling strategy for both accurate prediction and biologically meaningful interpretation.

### 2.3. Model Training Methods

The identification of biomarkers and interpretability analysis allows for the assessment of the contribution of model-derived features (particularly those embedded in the latent space) to drug response prediction. Key features identified through this process can subsequently be selected as candidate biomarkers for downstream survival risk analysis in patient cohorts. The VAE_LD model similarly utilizes this approach to pinpoint essential genes associated with drug resistance and sensitivity.

The final output layer of the VAE_LD model comprises two neurons, corresponding to the binary classification task of predicting drug sensitivity and resistance. For the classification task, the model employs the built-in multi-class cross-entropy loss function (torch.nn.CrossEntropyLoss) from the PyTorch library (v1.12.1). The mathematical expression for the multi-class cross-entropy loss is as follows:

(1) $$L=-\frac{1}{N}\sum_{i=1}^{N}\;\sum_{k=1}^{C}\;y_{ik}\log\left(p_{ik}\right)$$

where N is number of training samples (or of the current mini-batch). C is number of classes. $y_{ik}$ represent Ground-truth indicator, $y_{ik}=\;1$ if sample iii belongs to class k; otherwise $y_{ik}=\;0$ (one-hot encoding). $p_{ik}$ is predicted probability that sample i is in class k, with logits $z_{ik}$ $p_{ik}=\frac{e^{z_{ik}}}{\sum_{j=1}^{C}\;e^{z_{ij}}}$ (soft-max).

For the RF model, an ensemble of decision trees–node splits are determined by impurity minimization, specifically the Gini index, rather than by the conventional cross-entropy loss used in neural classifiers. This is represented as follows:

(2) $$Gini=1-\sum_{c=1}^{C}\;(p_{c}{)}^{2}$$

where $p_{c}$ is the proportion (empirical probability) of samples that belong to class c within the current node: $p_{c}=n_{c}/n$, where $n_{c}$ is the count of class ccc instances and n is the total number of instances in the node.

In contrast, XGB is trained under a binary logistic (cross-entropy) loss, optimising the log-likelihood of the two-class outcome at each boosting iteration.

(3) $$L\left(y_{i},{\hat{y}}_{i}\right)=-\left[y_{i}\log\left({\hat{y}}_{i}\right)+\left(1-y_{i}\right)\log\left(1-{\hat{y}}_{i}\right)\right]$$

where $y_{i}$ is ground-truth label for sample i; it takes the value 1 (positive class) or 0 (negative class). ${\hat{y}}_{i}$ is model-predicted probability that sample i belongs to the positive class: $0<{\hat{y}}_{i}<1$ (typically the sigmoid of a logit).

### 2.4. Model Evaluation Methods

Because the drug-response labels in the bulk RNA-seq data set are highly imbalanced, we adopted a k-nearest-neighbour over-sampling strategy, implemented via the Synthetic Minority Over-sampling Technique (SMOTE)—a core component of the imblearn package (version 0.12.3). The number of neighbours was kept at the default value of 5. In practice, SMOTE was applied to the Random-Forest (RF) and XGBoost (XGB) models, whereas the VAE_LD, VAE-LL and VAE-LS models leveraged a pre-trained scRNA-seq VAE to perform data augmentation.

For downstream drug-response prediction from RNA-seq profiles, we assessed model performance and generalisability using Accuracy, Recall (Sensitivity), Precision, F1-score, and the Matthews Correlation Coefficient (MCC). In addition, we plotted Receiver-Operating-Characteristic (ROC) curves and Precision–Recall (PRC) curves to visualise performance across the full range of classification thresholds.

### 2.5. Model Interpretation and Key Gene Identification Methods

For model interpretability and key-gene discovery, we applied Integrated Gradients (IG), GradientSHAP, and TreeSHAP to the network’s sensitivity and resistance prediction heads. Each method yields feature attribution scores that quantify, for every gene, its individual contribution to the sensitivity and resistance predictions, respectively, which are as follows:

(4) $$\begin{array}{cc} IG_{\mathrm{sensitivity},i}\left(x\right)= & \left(x_{i}-x_{i}^{\prime }\right)\times E_{x^{\prime }∼P\left(x^{\prime }\right)}\\ & \left[\int_{\alpha =0}^{1}\;\frac{\partial F_{\mathrm{sensitivity}}\left(x^{\prime }+\alpha \left(x-x^{\prime }\right)\right)}{\partial x_{i}}d\alpha \right] \end{array}$$

(5) $$\begin{array}{cc} IG_{\mathrm{resistance},i}\left(x\right)= & \left(x_{i}-x_{i}^{\prime }\right)\times E_{x^{\prime }∼P\left(x^{\prime }\right)}\\ & \left[\int_{\alpha =0}^{1}\;\frac{\partial F_{\mathrm{resistance}\;}\left(x^{\prime }+\alpha \left(x-x^{\prime }\right)\right)}{\partial x_{i}}d\alpha \right] \end{array}$$

(6) $$GS_{\mathrm{sensitivity},\;i}\left(x\right)=E_{x^{\prime }∼P\left(x^{\prime }\right)}\left[E_{\alpha ∼U\left(0,1\right)}\left[\left(x_{i}-x_{i}^{\prime }\right)\frac{\partial F_{\mathrm{sensitivity}}\left(x^{\prime }+\alpha \left(x-x^{\prime }\right)\right)}{\partial x_{i}}\right]\right]$$

(7) $$GS_{\mathrm{resistance},\;i}\left(x\right)=E_{x^{\prime }∼P\left(x^{\prime }\right)}\left[E_{\alpha ∼U\left(0,1\right)}\left[\left(x_{i}-x_{i}^{\prime }\right)\frac{\partial F_{\mathrm{resistance}}\left(x^{\prime }+\alpha \left(x-x^{\prime }\right)\right)}{\partial x_{i}}\right]\right]$$

where $F_{\mathrm{sensitivity}}$ represent the model output function for the sensitivity prediction head and $F_{\mathrm{resistance}}$ represent the resistance prediction head. x represents an input feature vector and $x^{\prime }$ represents a baseline input vector. In this experiment, $x^{\prime }$ is defined as the mean of x⁠. The feature $x_{i}$ denotes the i-th feature in the input vector.

(8) $$TS_{\mathrm{sensitivity},\;i}\left(x\right)=\sum_{t=1}^{T}\;\sum_{v\in V_{t,i}}\;\left(w_{t,v}\left(x\right)\left[f_{t,right}^{sensi}\left(v\right)-f_{t,left}^{sensi}\left(v\right)\right]\right)$$

(9) $$TS_{\mathrm{resistance},\;i}\left(x\right)=\sum_{t=1}^{T}\;\sum_{v\in V_{t,i}}\;\left(w_{t,v}\left(x\right)\left[f_{t,right}^{resi}\left(v\right)-f_{t,left}^{resi}\left(v\right)\right]\right)$$

where T is the total number of trees in the ensemble. $V_{t,i}$ denotes the set of internal nodes of tree t whose splitting feature is the i-th input feature $x_{i}$. $w_{t,v}(x)$ is the TreeSHAP weight of node v for the sample x: it encodes the Shapley coefficient that accounts for all coalitions of features that route x through node v. $f_{t,right}^{sensi}(v)$ and $f_{t,left}^{sensi}(v)$ are, respectively, the leaf-output values of the sensitivity prediction head that are reached when the path continues to the right or to the left child of node v in tree t; the term $f_{t,right}^{sensi}\left(v\right)-f_{t,left}^{sensi}(v)$ therefore measures the contribution of node v to the sensitivity prediction. Analogously, $f_{t,right}^{resi}(v)$ and $f_{t,left}^{resi}(v)$ are the corresponding leaf outputs of the resistance prediction head.

### 2.6. Survival Analysis

The survival-analysis module is designed to quantify how much the RNA-seq expression matrix contributes to the downstream drug-response prediction model. Genes with the highest absolute attribution scores obtained from IG, GradientSHAP and TreeSHAP are regarded as key genes for estimating patient survival risk. The expression levels of these key genes are then used to build a Cox proportional-hazards model and to generate the corresponding survival curves. Patients are stratified into high-risk and low-risk cohorts according to the median risk score, allowing direct comparison of survival outcomes between the two groups. The Cox proportional-hazards model is formulated as follows:

(10) $$h\left(t|X\right)=h_{0}\left(t\right)\exp\left(\beta^{⊤}X\right)$$

where t is the follow-up time. $X=(x_{1},x_{2},\ldots ,x_{p}{)}^{⊤}$, the selected p gene expression vectors.

h(t∣X) represents the instantaneous hazard rate of the individual at time t. $h_{0}(t)$ is the baseline hazard function (independent of covariates). $\beta =(\beta_{1},\beta_{2},\ldots ,\beta_{p}{)}^{⊤}$ represents the estimated covariate coefficient.

(11) $$S\left(t|X\right)=\mathrm{Pr}\left(T>t|X\right)=\exp\left(-\int_{0}^{t}\;h\left(u|X\right)du\right)={\left[S_{0}\left(t\right)\right]}^{\exp\left(\beta^{⊤}X\right)}$$

where S(t∣X) represents the probability of an individual surviving before time t.

$S_{0}(t)=exp[-\int_{0}^{t}\;h_{0}(u)du]$ is the baseline survival function. In $exp(\beta^{⊤}X)>1$, if the prognosis is poor, the curve shifts downward; otherwise, it shifts upward.

(12) $$l\left(\beta \right)=\sum_{i=1}^{n}\;\delta_{i}\left[\beta^{⊤}X_{i}-\log\left(\sum_{j:t_{j}\geq t_{i}}\;\exp\left(\beta^{⊤}X_{j}\right)\right)\right]$$

where n is the sample size; $t_{i}$ represents the event or censoring time of the i-th patient. $\delta_{i}=1$ represents the occurrence of an event, and $\delta_{i}=0$ represents censoring. $R(t_{i})=\{j|t_{j}\geq t_{i}\}$ represents the risk set—all individuals who have not experienced an event at ti. Finally, $\hat{\beta }$ is obtained by maximizing l(β), and the standard error and 95% confidence interval are estimated by Hessian.

### 2.7. The Entire Process of Data Analysis

**Figure 1:**
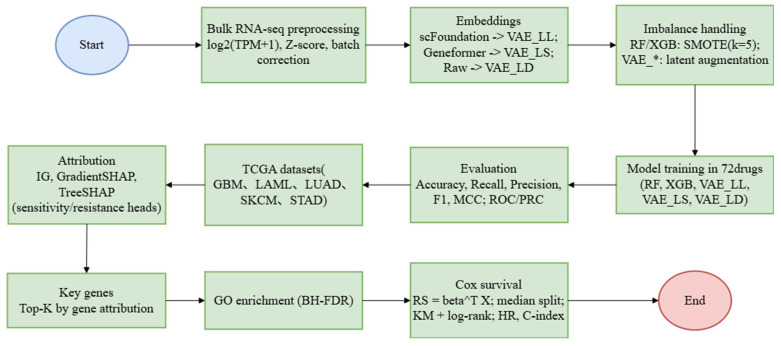
End-to-end analysis workflow covering RNA-seq preprocessing, representation learning, imbalance handling, multi-model training and evaluation across 72 drugs, attribution-based key-gene nomination, GO enrichment, and survival validation on TCGA cohorts. (The workflow comprises three stages: data preprocessing and feature engineering, model training and evaluation, and model interpretability with biological validation. In the preprocessing stage, bulk RNA-seq data are normalized using log2(TPM+1) transformation and Z-score scaling, followed by batch correction to minimize technical noise. To capture nonlinear gene–gene relationships, three VAE-based embedding strategies are explored to generate low-dimensional features for drug-response prediction. VAE_LD serves as a baseline trained by knowledge distillation directly on the preprocessed expression matrix. VAE_LL leverages latent representations extracted by the single-cell pretrained model scFoundation as inputs to the VAE. VAE_LS uses latent representations derived from the pretrained model Geneformer as inputs to the VAE. In the modeling stage, predictive models are built and benchmarked for 72 drugs. Class imbalance is addressed with SMOTE for the classical machine-learning models (random forest and XGBoost), whereas latent-space augmentation is applied for the VAE variants. Performance is assessed comprehensively using accuracy, F1-score, Matthews correlation coefficient, and area under the ROC and PR curves. In the interpretability and validation stage, attribution methods including Integrated Gradients, GradientSHAP, and TreeSHAP are applied on TCGA to identify the top-K genes that most strongly drive predictions of sensitivity or resistance. These genes are subjected to Gene Ontology enrichment to elucidate core biological functions. Clinical relevance is then evaluated with Cox proportional-hazards modeling to compute a risk score, together with Kaplan–Meier curves, log-rank testing, and the concordance index, thereby assessing the prognostic value of the identified genes).

The workflow is summarized in Figure 1. Bulk RNA-seq profiles were first preprocessed with log_2_(TPM + 1) transformation, Z-score normalization, and batch correction. Three representation routes then produced embeddings: scFoundation-derived features were fed to VAE_LL, Geneformer features to VAE_LS, and raw expression to VAE_LD. Class imbalance was addressed with SMOTE for tree-based learners and latent-space augmentation for VAE models. Supervised training across 72 drugs used Random Forest, XGBoost, and the three VAE variants, with performance assessed by accuracy, recall, precision, F1, MCC, and ROC/PRC curves on TCGA cohorts spanning GBM, LAML, LUAD, SKCM, and STAD. Model explainability with Integrated Gradients, GradientSHAP, and TreeSHAP generated gene-level attributions from the sensitivity and resistance heads, from which top-ranked markers were nominated. These candidates underwent GO enrichment with Benjamini–Hochberg FDR control, and clinical relevance was tested by Cox proportional hazards and median-split Kaplan–Meier analyses with log-rank statistics, reporting hazard ratios and C-index.

#### 2.7.1. Bulk RNA-Seq Download from GDSC

We downloaded the RNA-seq gene-expression dataset in h5ad format containing unnormalized raw counts. The data were processed into a matrix in which rows represent samples (cell lines) and columns represent genes (using Ensembl IDs). Because gene names in GDSC are stored as gene symbols, we developed a utility to convert gene symbols to Ensembl IDs. We then constructed the mapping from RNA-seq profiles to the drug-response labels required for model transfer, providing the supervision for learning.

We first normalized the GDSC expression matrix: TPM values were transformed as log_2_(TPM + 1) and then z-score standardized per gene so that each gene had mean 0 and variance 1 across all samples.

$${\tilde{x}}_{ij}=\log_{2}(x_{ij}+1),\;z_{ij}=\frac{{\tilde{x}}_{ij}-\mu_{j}}{\sigma_{j}+\epsilon },$$

#### 2.7.2. BioMart-Based Mapping and Batch Correction

BioMart-exported mapping files were used to convert Ensembl IDs (with version numbers/suffixes removed) to HGNC gene symbols; duplicated symbols were aggregated by taking the mean expression, genes missing in a matrix were imputed as 0, and column order was harmonized. When explicit batch labels were available, batch effects were corrected with ComBat under an empirical Bayes framework:

$$x_{ij}=\alpha_{j}+\beta_{b(i),j}+\gamma_{b(i),j}\epsilon_{ij},$$

#### 2.7.3. Bulk RNA-Seq Feature Embeddings

Pretrained expression models, scFoundation and Geneformer, were used as base encoders to derive embeddings from bulk RNA-seq profiles.

#### 2.7.4. Drug-Response Binarization Threshold

Continuous GDSC response metrics were converted to binary labels. Using AUC as an example, a threshold of 0.5 was applied (AUC ≤ 0.5 labeled sensitive; otherwise resistant).

#### 2.7.5. Full Hyperparameter Settings

For VAE_LL and VAE_LS, we used batch_size = 64, learning_rate = 2 × 10^−5^, weight_decay = 3 × 10^−3^, and num_epochs = 150. The small learning rate stabilizes fine-tuning of pretrained embeddings; weight decay provides L2 regularization to limit overfitting in high-dimensional features; 150 epochs ensure convergence; batch_size = 64 balances compute with gradient stability. VAE_LD used the same learning rate, regularization, and number of epochs, with batch_size increased to 128 to improve gradient-estimation stability and throughput when operating directly on bulk expression matrices. For tree-based models, Random Forest (RF) used n_estimators = 100, max_depth = 10, min_samples_split = 2, min_samples_leaf = 1, and max_features = 0.8; a medium-sized forest and limited depth reduce variance, feature subsampling (max_features = 0.8) decorrelates trees and improves generalization, and the split/leaf settings maintain adequate samples per leaf. XGBoost was configured with n_estimators = 100, max_depth = 6, learning_rate = 0.1, subsample = 1.0, and colsample_bytree = 1.0. With a relatively shallow depth and moderate step size, this strikes a bias–variance trade-off; given the dataset size, row and column subsampling were not enabled so as to leverage all available samples and features.

#### 2.7.6. Knowledge Distillation for VAE_LD

VAE_LD builds on scATD-sf-dist trained on the Panglao single-cell dataset. Optimization minimizes a reconstruction loss (REC), a Kullback–Leibler divergence loss (KLD), and an additional cosine-similarity–based distillation loss (COD). The COD term measures the cosine similarity between the teacher latent vector fTf_T produced by the scATD-sf model and the student latent vector fSf_S produced by scATD-sf-dist for the same cell, thereby encouraging alignment of their embeddings.

$$L_{COD}=1-\frac{f_{T}\cdot f_{S}}{\left\|f_{T}\right\|\left\|f_{S}\right\|}$$

Total loss function

$$L_{total}=\beta \cdot L_{REC}+L_{KLD}+\theta \cdot L_{COD}$$

where θ is a hyperparameter that trades off distillation and reconstruction; β, analogous to the coefficient used in Res-VAE, was fixed at 1000 in this study; and γ was tuned with Optuna together with network depth and other architectural hyperparameters.

#### 2.7.7. Model Training

VAE_LL used embeddings derived from scFoundation as input, whereas VAE_LS used embeddings derived from Geneformer. The preprocessed bulk matrices were provided as inputs to VAE_LD, RF, and XGBoost. Drug-sensitivity prediction was performed for all 72 agents. Five-fold cross-validation was used for pretraining and model checkpoints were saved; the fold achieving the best AUC was selected as the checkpoint for downstream analyses.

#### 2.7.8. TCGA Data Acquisition

RNA-seq profiles and corresponding clinical outcome data were retrieved from The Cancer Genome Atlas (TCGA) across five cancer types: glioblastoma (GBM), acute myeloid leukemia (LAML), lung adenocarcinoma (LUAD), skin cutaneous melanoma (SKCM), and stomach adenocarcinoma (STAD).

#### 2.7.9. TCGA Preprocessing

TCGA data were processed into a matrix with rows as samples and columns as genes (Ensembl IDs). Gene symbols were converted to Ensembl IDs using our mapping utility. A mapping from RNA-seq profiles to the drug-response labels required for transfer was constructed to supply supervision, and nine clinically relevant drugs were selected from the literature for drug-sensitivity modeling.

#### 2.7.10. Downstream Analyses on TCGA

VAE_LL, VAE_LS, and VAE_LD employed Integrated Gradients (IG) and GradientSHAP to identify biomarkers, using the per-gene mean as the baseline for both methods. RF and XGBoost used TreeSHAP for attribution. Genes were ranked by attribution score in descending order, the top K (K = 100) were retained for each method, and the intersection and union were computed across the three interpretability families.

Prognostic risk was evaluated with a Cox proportional hazards model using the expression of key genes as covariates *X*. The hazard and partial log-likelihood were specified as

$$(t|X)=h_{0}(t)\exp(\beta^{⊤}X),\;l(\beta )=\sum_{i}\delta_{i}\left[\beta^{⊤}X_{i}-\log\sum_{j:t_{j}\geq t_{i}}e^{\beta^{⊤}X_{j}}\right]$$

Individual risk scores were computed as ${RS}_{i}={\hat{\beta }}^{⊤}X_{i}$ and patients were dichotomized at the median into high- and low-risk groups. Kaplan–Meier curves were plotted and compared by the log-rank test. The proportional hazards assumption was examined using Schoenfeld residuals; if violated, stratified Cox models or time-by-covariate interactions were employed. We report hazard ratios (HRs) with 95% confidence intervals, the concordance index (C-index), and time-dependent AUCs at 1, 3, and 5 years.

## 3. Results

We first benchmark the five models on pharmacogenomic cell-line data to establish predictive performance and feature stability. We then extend the best performers to TCGA cohorts to explore cross-tumor applicability and to generate biomarker hypotheses in clinically relevant settings. Finally, we interrogate tumor- and drug-specific mechanisms through interpretability, network analyses, pathway enrichment, and prognostic stratification.

### 3.1. Drug Resistance Prediction and Key Gene Identification in Cell Lines

This subsection evaluates whether residual VAE variants and ensemble learners can accurately classify resistance in vitro and yield stable feature attributions. We use five-fold cross-validation on GDSC to compare discrimination, calibration, and robustness across drugs, and we examine concordance of gene-level importance between deep and classical models.

To systematically evaluate the performance of different machine learning frameworks in predicting tumor drug resistance, we employed five-fold cross-validation on the GDSC cell line drug sensitivity dataset. The dataset was split into training and validation sets, and the performance of five models (VAE_LD, VAE_LS, VAE_LL, RF, and XGB) was compared using six evaluation metrics: AUC, MCC, F1-score, recall, accuracy, and precision.

**Figure 2:**
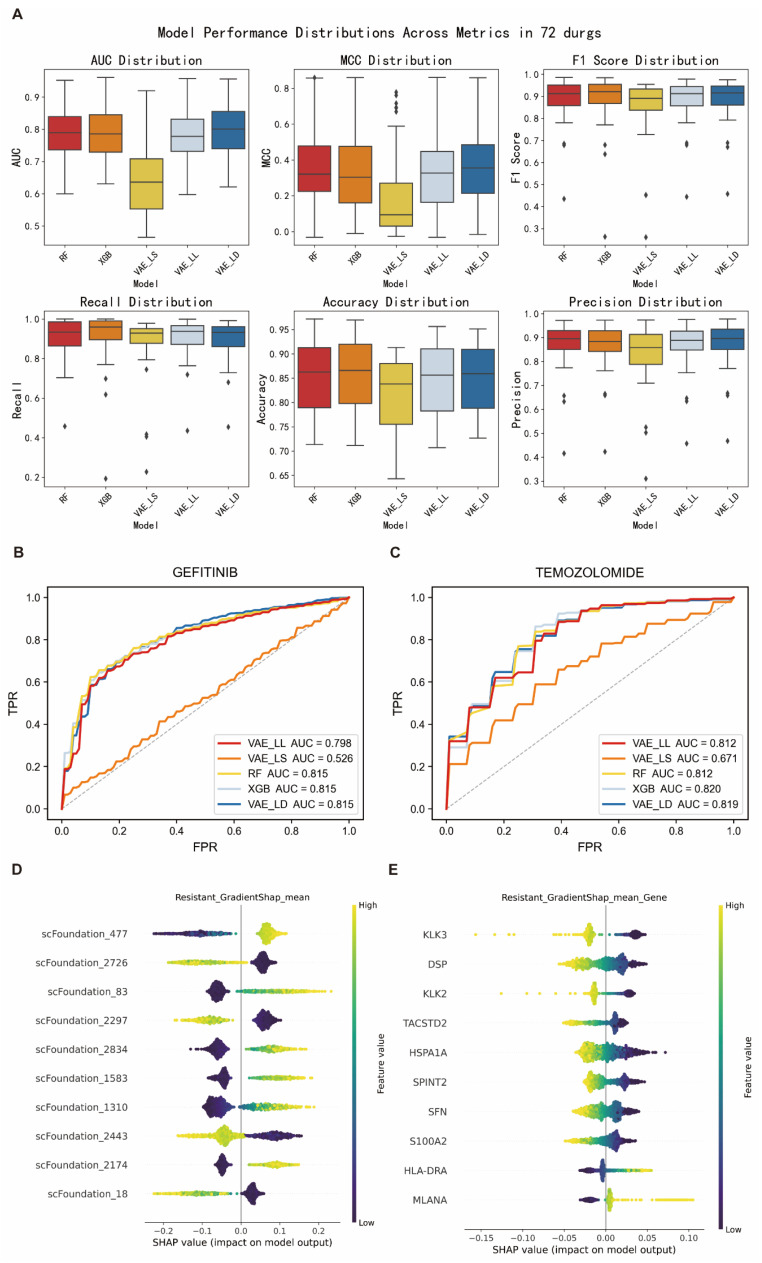
Performance and Interpretability of the Proposed Models on the GDSC Dataset. (**A**): Box plot of the model’s prediction of the evaluation matrix of 72 drugs. (**B**): ROC plot of GEFITINIB’s 5-fold inference on the Bulk dataset. (**C**): ROC plot of TEMOZOLOMIDE’s 5-fold inference on the Bulk dataset. (**D**): Visualization of top important features extracted from Bulk transcriptome datasets by the scFoundation foundation model, interpreted by GradientSHAP for the Resistant (left) and Sensitive (right) drug response groups. Each dot represents a single sample, with color indicating the original feature value (from low [purple] to high [yellow]). The *x*-axis denotes the SHAP value, reflecting the impact (direction and magnitude) of each feature on the model output. (**E**): Visualization of feature importance at the gene expression level using the same interpretability methods, respectively, for Resistant and Sensitive groups. The top important genes are listed, showing their contribution to model prediction and distribution of feature values.

The results showed that VAE_LD outperformed all other models across all metrics, achieving an average AUC of 0.81, MCC of 0.37, F1-score of 0.89, recall of 0.91, accuracy of 0.86, and precision of 0.88 (Figure 2A, Table 1). It also demonstrated stable performance across nine commonly used anticancer drugs (Table 2 and Supplementary Figure S1). Furthermore, VAE_LD consistently achieved the highest average AUC across individual drugs (Figure 2B,C). The two traditional machine learning models, RF and XGB, also exhibited comparable and robust performance. In contrast, the conventional deep learning model VAE_LS performed significantly worse across all evaluation metrics, with a median AUC of only 0.65, suggesting its limited ability to capture key features related to drug resistance. Consequently, VAE_LS was excluded from further analysis, and VAE_LD was selected as the primary model for subsequent interpretability studies.

Given that the VAE_LD model does not support direct feature attribution, we employed its backbone architecture, VAE_LL, to perform feature-level interpretability analysis using IG and GradientSHAP algorithms. In the context of gefitinib resistance prediction, the latent feature “scFoundation_477” was consistently identified as the most important positive predictor, while “scFoundation_2726” showed a strong negative correlation with resistance, it was markedly reduced in resistant samples and elevated in sensitive ones (Figure 2D and Supplementary Figure S2). This directional consistency supports the biological relevance of the model-learned features.

To further interpret these latent features, we mapped them back to specific genes and identified three key genes: *KLK3*, *TACSTD2*, and *PAGE4*. Previous studies have shown that *KLK3* plays a critical role in inhibiting prostate cancer progression and restoring chemotherapy sensitivity [19], while *PAGE4* has been reported to significantly enhance tumor cell responsiveness to gefitinib and other therapies [20] (Figure 2E and Supplementary Figure S2). These three genes were consistently ranked as the most important across both IG and GradientSHAP analyses, reinforcing the robustness of the model’s interpretability.

To explore the biological context of these key genes, we conducted GO and KEGG pathway enrichment analyses. The results revealed that DSP, KRT19, and members of the S100 family were predominantly enriched in pathways related to epithelial–mesenchymal transition (EMT) [21], cell adhesion, and migration, while HSPA1A and SFN were enriched in apoptosis-related pathways [22]. Additional significantly enriched pathways included those related to angiogenesis, lymphangiogenesis, and Wnt signaling regulation, all of which are highly relevant to the development of drug resistance.

The VAE_LD model not only demonstrated superior predictive performance in drug resistance modeling but also enabled the identification of biologically meaningful features and key regulatory genes through interpretability analysis. These findings create a closed loop among molecular mechanisms, model attributions, and potential translational applications, providing a solid foundation for further exploration of tumor heterogeneity and personalized cancer treatment strategies.

### 3.2. Identification of Drug Sensitivity Biomarkers Across Multiple Cancers in a Clinical Prognostic Context

To examine clinical relevance, we transferred the best-performing models to TCGA cohorts spanning five tumor types. Here we assess whether gene signatures prioritized in cell lines retain signal in patient transcriptomes and whether they stratify prognosis, recognizing that these analyses are exploratory in the absence of direct treatment-response data.

In clinical applications, the identification of robust biomarkers is essential for elucidating cancer heterogeneity and evaluating the translational potential of predictive models. Our previous analyses were primarily conducted using the GDSC dataset, which provides drug sensitivity data derived from cancer cell lines. While this resource is valuable for model training and mechanistic exploration, it lacks critical components such as the tumor microenvironment, thereby limiting its ability to fully capture actual drug responses observed in patients.

To address this limitation, we incorporated data from The Cancer Genome Atlas (TCGA), which more accurately reflects clinical reality, particularly in terms of patient heterogeneity and its relevance to precision medicine. VAE_LD demonstrated strong predictive performance and interpretability on the GDSC training set. To further assess its predictive capability in clinically relevant settings, we applied the model to RNA-Seq data from five cancer types in the TCGA cohort: GBM (*n* = 175), LUAD (*n* = 589), LAML (*n* = 151), SKCM (*n* = 473), and STAD (*n* = 448).

**Figure 3:**
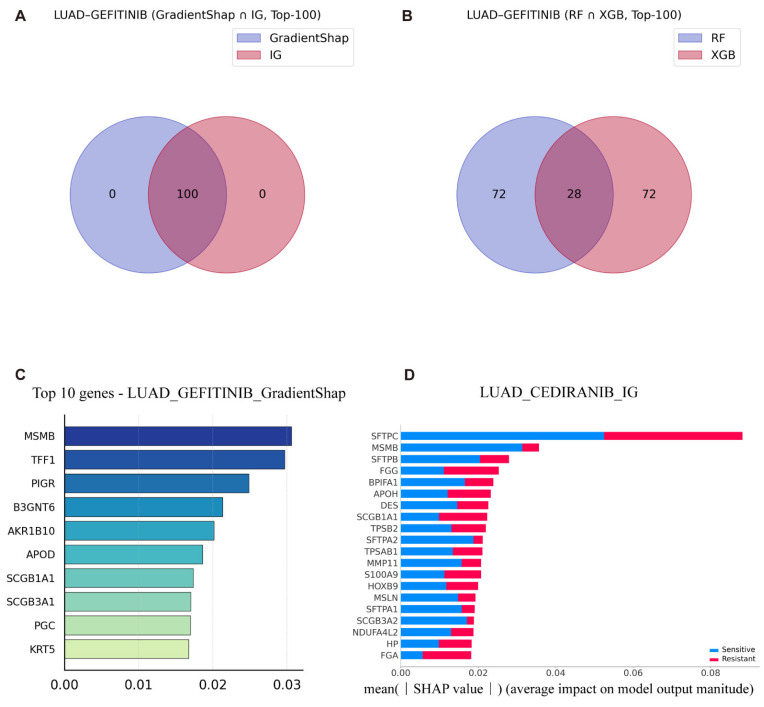
Interpretability analysis of key genes in LUAD Gefitinib/Cediranib sensitivity models. (**A**): Gene intersection diagram of the T100 genes selected based on IG and GradientSHAP explainable methods (**B**): Gene intersection diagram of the T100 genes selected based on RF and XGB interpretable methods. (**C**): Bar plots show the top 10 genes ranked by mean (|SHAP value|) for Gefitinib response prediction in the TCGA-LUAD cohort, using GradientSHAP. The x-axis indicates the average contribution of each gene to the model output. Blue and red bars represent feature importance in resistant and sensitive subgroups, respectively. (**D**): Similarly, the top 20 genes for Cediranib sensitivity prediction are displayed (GradientSHAP), with blue and red bars denoting importance in sensitive and resistant subgroups.

To maintain consistency with our prior predictive analyses, we focused in particular on LUAD patient data from TCGA to investigate the molecular mechanisms underlying gefitinib resistance. This approach enhances the clinical relevance of the identified biomarkers and supports their potential utility in guiding individualized therapeutic strategies. We first used the intersection of the T100 genes selected based on different interpretability methods to analyze gefitinib-related drug resistance in LUAD patients (Figure 3A,B).

Gefitinib is most commonly used for the treatment of EGFR-mutant LUAD patients. By comparing the interpretability results of the VAE_LD model using IG and GradientSHAP with those of traditional machine learning models (RF with TreeSHAP, and XGB), we identified key genes implicated in drug resistance, including *TFF1* and *B3GNT6* (Figure 3C and See Supplementary Figures S3 and S4 for details).

Previous studies have reported that elevated expression of *TFF1* promotes cell proliferation and survival in LUAD and is significantly associated with shorter overall survival [23]. Consistently, both IG and GradientSHAP interpretability analyses indicated that high expression of *TFF1* (highlighted in yellow for strong enrichment) positively contributes to gefitinib resistance. Similarly, *B3GNT6* was also identified by both interpretability methods as a strong positive regulator of resistance. Huang et al. previously demonstrated that elevated *B3GNT6* expression is closely linked to LUAD progression [24]. In patients treated with EGFR-TKIs, it has been shown that TKIs modulate the MUC1 glycosylation axis, in which *B3GNT6* acts as a key regulator. MUC1 glycan isomerization has been implicated in altering EGFR recycling and promoting immune evasion, thereby contributing to the development of a drug-resistant tumor microenvironment. Notably, elevated expression of *B3GNT6* is a hallmark of this glycan isomerization process, directly supporting VAE_LD model’s prediction that *B3GNT6* promotes gefitinib resistance in LUAD patients.

Although machine learning models have demonstrated high predictive accuracy in previous studies, whether their interpretability matches that of deep learning models remains unclear. To address this, we performed an interpretability analysis using XGB and RF in combination with TreeSHAP. Interestingly, the results yielded contradictory conclusions compared to those from deep learning models, and this inconsistency was observed across multiple datasets (See Supplementary Figures S3 and S4 for details). In the context of resistance prediction, *IRF6* emerged as a key gene. While previous studies have shown that *IRF6* suppression contributes to acquired drug resistance in tumors [25], TreeSHAP attributed a role for *IRF6* that contradicted these findings. This discrepancy highlights the potential limitations of interpretability in machine learning models, when applied to complex biological datasets. These results underscore the need for caution when relying solely on traditional interpretability tools in high-dimensional, biologically heterogeneous contexts.

Additionally, to explore drug resistance beyond gefitinib in LUAD, we analyzed resistance mechanisms associated with cediranib. Both IG and GradientSHAP identified key genes involved in cediranib resistance, including *SFTPC*, *FGG*, *AZGP1*, and *FGA* (Figure 3D and See Supplementary Figures S3 and S4 for details). Previous studies have shown that low expression of *SFTPC* promotes cell proliferation and epithelial–mesenchymal transition (EMT) in LUAD and is associated with shorter overall survival [26]. However, both IG and GradientSHAP analyses indicated that high expression of *SFTPC* (visualized as yellow for high enrichment) positively regulates cediranib resistance. This finding is particularly intriguing. Deeper mechanistic studies revealed that *SFTPC* is a marker of alveolar type II (AT2) cell differentiation, and its high expression suggests that tumors retain features of the TRU subtype [27]. TRU-type tumors typically exhibit high vascular maturity, low microvessel density, and reduced dependency on VEGF-A. Given that cediranib primarily targets VEGFR-1/2/3, tumors with these characteristics may develop intrinsic resistance. Thus, high *SFTPC* expression may promote cediranib resistance in LUAD, even in patients with otherwise favorable prognostic features. This underscores that genes associated with good prognosis do not necessarily imply drug sensitivity and must be interpreted within the context of specific therapeutic mechanisms.

Similarly, both interpretability methods identified *FGA* as a strong positive regulator of cediranib resistance. Shang et al. previously reported that high *FGA* expression in EGFR-mutant LUAD is negatively correlated with chemotherapy response, directly supporting our findings that *FGA* contributes to cediranib resistance [28]. In the cediranib sensitivity analysis, both IG and GradientSHAP further confirmed that high *SFTPC* expression negatively regulates drug sensitivity, providing additional validation of the model’s accuracy. Both interpretability approaches consistently ranked *SFTPC* among the top 20 most important genes, with low expression favoring drug sensitivity and high expression promoting resistance.

Comprehensive analyses for other cancer types and their associated drugs are included in the Supplementary Materials and are not discussed in detail here. (See Supplementary Figures S3 and S4 for details).

### 3.3. Biomarker Analysis for Temozolomide Response in GBM

As a focused exemplar, we analyze temozolomide in GBM to link model predictions with gene-level mechanisms. We integrate attributions from deep and classical models to highlight convergent and divergent biomarkers and to position these signals within the GBM literature.

Biomarkers play a critical role in predicting tumor response to treatment, and the identification of effective biomarkers enables the accurate selection of appropriate treatment candidates in advance. In this study, the deep learning model VAE_LD was integrated with two interpretability algorithms, IG and GradientSHAP. These algorithms operate directly on gene expression data and provide joint visualization of predictive results and the contributing genes. In addition, the simultaneous presentation of two traditional machine learning models further enabled a comparative evaluation of their performance in predicting tumor drug resistance and in generating interpretable outputs. This integrative framework provides a robust approach for biomarker discovery and enhances the clinical applicability of predictive models in oncology.

**Figure 4:**
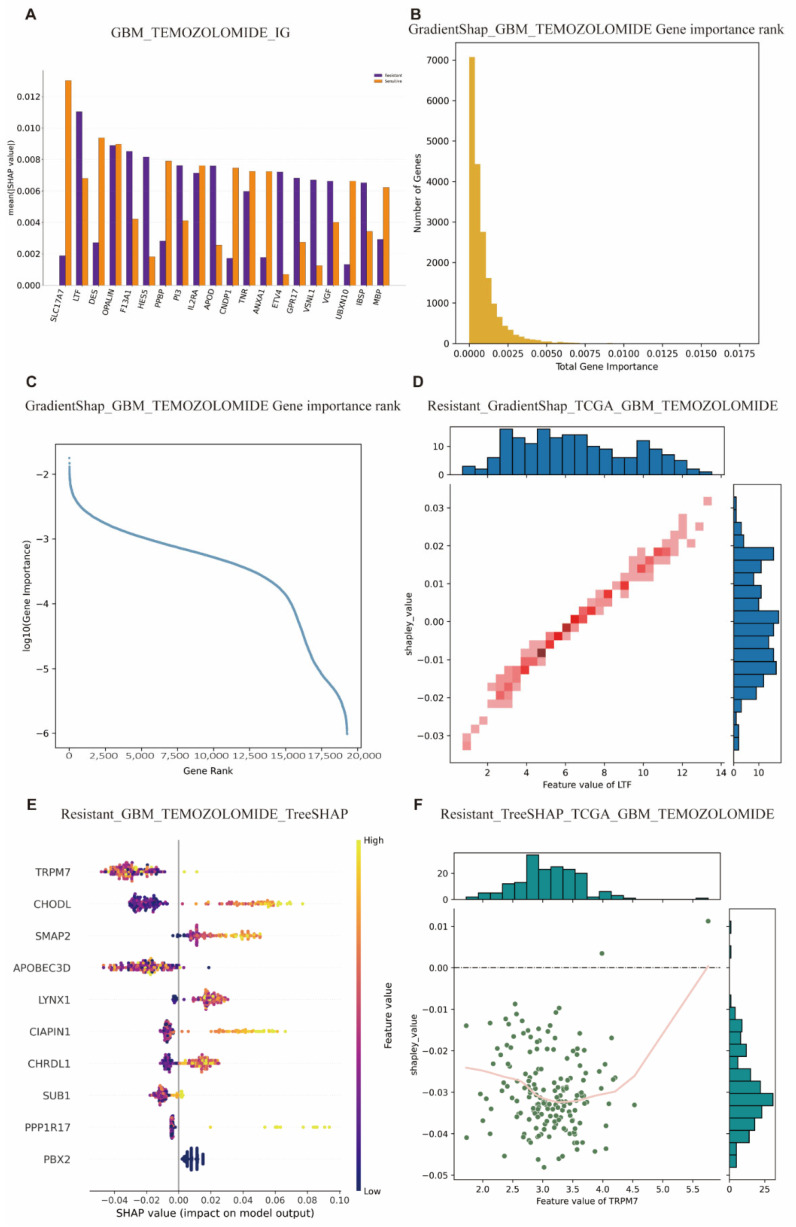
Interpretability analysis of the GBM Temozolomide sensitivity prediction model using SHAP-based algorithms. (**A**) Top 20 important genes ranked by mean absolute SHAP values (mean (|SHAP value|)), visualized by GradientSHAP. Blue and red bars indicate sensitive and resistant subgroups, respectively. (**B**,**C**) Visualization of genome-wide feature importance using GradientSHAP. (**B**) Histogram of total gene importance; (**C**) log-log plot showing the power-law distribution of gene importance by rank. (**D**) Relationship between feature values and Shapley values for representative genes (*LTF* in resistant group) using GradientSHAP. Marginal distributions of feature values and SHAP values are shown at the top and right. Blue indicates the frequency distribution of the data. The top blue histogram shows the distribution of the x-axis feature “Feature value of *LTF*” (expression level of the *LTF* gene), where the height of each bar represents the number of samples falling within that feature-value bin. The right blue histogram shows the distribution of the y-axis variable “shapley_value” (SHAP value), where the length of each bar represents the number of samples within that value range. Red encodes the density or concentration of data points. The central red scatterplot displays the relationship between the *LTF* feature values and their corresponding SHAP values, with each dot representing one sample. Color intensity reflects local point density: darker red indicates a higher concentration of samples. (**E**) Top 10 gene importance ranking by mean (|SHAP value|) using TreeSHAP, colored by subgroup (blue: sensitive, red: resistant). (**F**) Feature value–Shapley value relationship for *TRPM7* in the resistant group under TreeSHAP.

Temozolomide (TMZ) is a first-line chemotherapeutic agent for GBM [29]. Using this drug as an example, we conducted an in-depth analysis of predictive biomarkers in GBM patients treated with TMZ from the TCGA database, utilizing the aforementioned models and interpretability algorithms. Both VAE_LD and IG/GradientSHAP identified *OPALIN*, *LTF*, and *IL2RA* as the most important genes (Figure 4A). These genes ranked 3/19,264, 9/19,264, and 5/19,264, respectively, in the resistance prediction group, and 2/17,006, 8/17,006, and 1/17,006 in the sensitivity prediction group (Figure 4B,C, Table 3). Among these, *OPALIN* was consistently ranked highest and showed a strong degree of symmetry across both resistance and sensitivity predictions.

Previous studies have shown that *OPALIN* is highly enriched in adult brain tissue and is primarily involved in oligodendrocyte differentiation [30]. However, its role in GBM remains poorly characterized. One study reported a significant association between *OPALIN* expression and decreased Karnofsky Performance Status (KPS) scores in elderly GBM patients, while no such association was found in younger patients. This suggests that *OPALIN* may not directly mediate drug resistance, but instead may reflect cell differentiation status in the brain.

In contrast, *LTF* displayed a more asymmetric pattern, with stronger contributions to resistance predictions (Figure 4D). Prior research has demonstrated a significant positive correlation between *LTF* overexpression and poor prognosis in GBM patients, as well as a strong association with immune evasion, thus supporting the biological relevance and accuracy of the model’s predictions [31].

Additionally, *SLC17A7* emerged as a top-ranked gene in the sensitivity group (Figure 4E), consistent with previous findings. *SLC17A7* is considered a tumor suppressor, and its overexpression has been shown to inhibit GBM cell proliferation and invasion [32].

Since TreeSHAP and XGB are based on binary classification frameworks, their interpretability outputs are generally symmetric. The RF model combined with TreeSHAP identified *TRPM7*, *CHODL*, and *SMAP2* as the most important genes (See Supplementary Figures S3–S7 for details). In the context of drug resistance, *TRPM7* was suggested by TreeSHAP to be lowly expressed in GBM and associated with resistance alleviation (Figure 4F). However, this finding is inconsistent with prior studies. Liu et al. reported that *TRPM7* is highly expressed in GBM and promotes both proliferation and resistance, primarily by upregulating tumor-associated stem cell markers [33]. Consistent with this, the XGB model identified *TRPM7* as highly expressed and positively associated with drug resistance (See Supplementary Figures S3–S7 for details). Comprehensive analyses of other cancer types and their corresponding drugs are included in the Supplementary Materials and are not discussed in detail here. (See Supplementary Figures S3–S7 for details).

### 3.4. Functional Network and Pathway Analysis of Temozolomide-Associated Biomarkers

To contextualize candidate genes, we map them onto interaction networks and biological processes. We test whether pathways implicated by interpretability methods converge on coherent programs related to angiogenesis, immune trafficking, and neural–tumor interactions that may underlie resistance.

**Figure 5:**
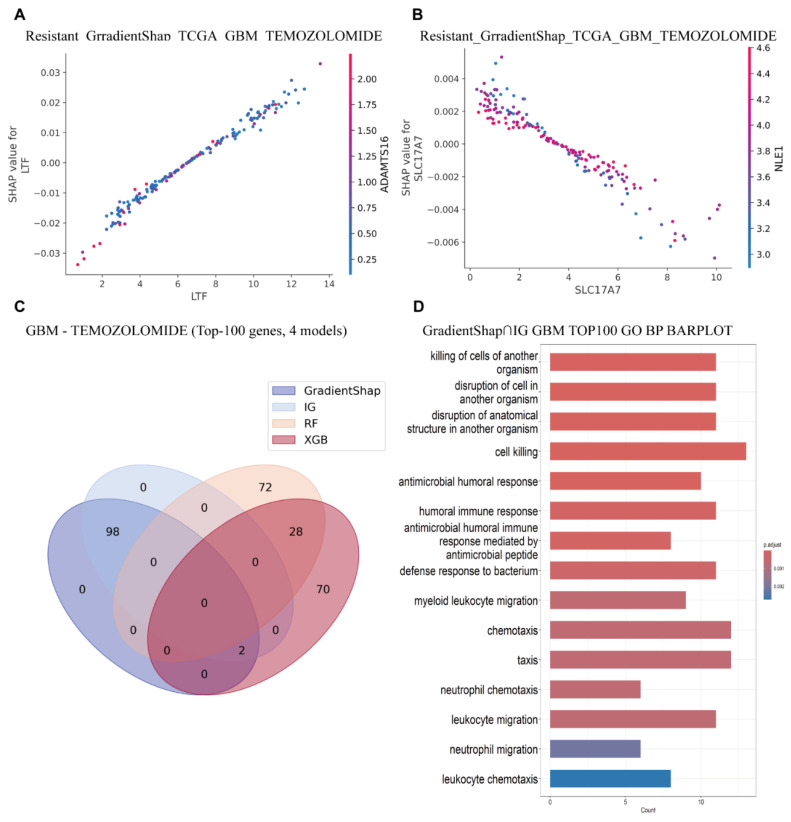
GBM Temozolomide resistance feature interactions and GO enrichment analysis based on multiple interpretability methods. (**A**) Scatter plots showing the relationship between *LTF* feature values and their SHAP values, derived from GradientSHAP, respectively. Dot color indicates the expression level of *ADAMTS16*. (**B**) Scatter plots for *SLC17A7* feature values and their SHAP values, based on GradientSHAP. Dot color denotes the expression of *NLE1*. (**C**) Gene intersection diagram of the top 100 important genes identified based on different models (**D**) Bar plot of GO Biological Process enrichment for the top 100 important genes identified by GradientSHAP and Integrated Gradients. Bar color indicates adjusted *p*-value (*p*.adjust), length represents gene count per GO term.

To further investigate the role of key genes in gene interaction networks and pathway enrichment, we employed the VAE_LD model in combination with IG/GradientSHAP and conventional machine learning algorithms. The four most important genes identified in the previous analysis were selected for gene interaction analysis. This revealed a particularly strong interaction between *LTF* and *ADAMTS16*, which appear to cooperatively contribute to GBM drug resistance (Figure 5A and See Supplementary Figures S8–S10 for details). *ADAMTS16* has been previously shown to drive epithelial–mesenchymal transition (EMT) and metastasis in various cancers, ultimately leading to the development of drug resistance [34].

In addition, we observed an antagonistic relationship between *SLC17A7* and *NLE1*, wherein high expression of *NLE1* appears to suppress *SLC17A7*, thereby contributing to chemotherapy resistance (Figure 5B and See Supplementary Figures S8–S10 for details). Interestingly, previous studies have reported that *NLE1* is a critical regulator of brain tumor stem cell growth and survival in GBM. Targeting *NLE1* has been shown to inhibit stemness features and restore the sensitivity of GBM cells to radiotherapy [35]. These findings suggest that elevated *NLE1* expression may downregulate *SLC17A7*, thus promoting treatment resistance.

Based on the intersection of T100 genes selected by four interpretable methods, it was found that the T100 genes selected by the IG/GradientSHAP interpretable method were completely consistent. However, there were significant differences between the T100 genes selected by the two machine learning models (Figure 5C).

Furthermore, to elucidate potential mechanisms of action, we selected the top 100 most important genes based on the VAE_LD model combined with IG and GradientSHAP, and conducted GO pathway enrichment analyses. GO enrichment results from both interpretability algorithms revealed consistent enrichment in pathways related to cell killing/antimicrobial defense, granulocyte/myeloid chemotaxis, synaptic vesicle endocytosis, and glial cell development. These findings suggest that necrosis- and inflammation-driven innate immune storms, in conjunction with neuro-tumor interactions, are prevalent in GBM, and are associated with poor prognosis, radio/chemotherapy resistance, and an immunosuppressive microenvironment (Figure 5D). This insight supports several promising therapeutic strategies, such as CXCL8-CXCR2 axis inhibition, disruption of tumor–neuron synapses, and targeting of reactivated developmental pathways [36].

Moreover, intersecting the most important genes identified by both interpretability algorithms yielded highly consistent enrichment patterns, particularly in cell killing and granulocyte/myeloid chemotaxis, further confirming their association with treatment resistance and adverse clinical outcomes.

In contrast, the two machine learning models produced differing enrichment profiles. The XGB algorithm primarily highlighted pathways associated with RNA/DNA editing, viral replication inhibition, vascular endothelial activation, neuronal dendritic self-avoidance, and immune enhancement. These features suggest that the corresponding patient subtype may be more responsive to interferon-based adjuvant therapies, oncolytic viruses, anti-angiogenic therapies, and immune checkpoint inhibitors, although they differ substantially from the pathways expected based on prior knowledge (See Supplementary Figures S8–S10 for details). The TreeSHAP algorithm identified additional pathways, including Rac-GTPase signaling and cytoskeletal remodeling, postsynaptic structure assembly, and neuronal coupling, which are plausibly linked to drug resistance mechanisms (See Supplementary Figures S8–S10 for details).

Overall, these findings suggest that the VAE_LD deep learning model, in combination with interpretable algorithms, provides superior accuracy in pathway-level enrichment analysis compared to traditional machine learning approaches. Analyses for additional tumor types and drug response patterns are provided in the Supplementary Materials. (See Supplementary Figures S8–S10 for details).

### 3.5. Prognostic Risk Analysis in GBM Patients Based on Gene Biomarkers

The next analysis evaluates whether gene sets derived from model attributions stratify overall survival in GBM. Compact and extended panels are compared to balance statistical power with clinical feasibility and to assess the portability of transcriptomic risk scores.

**Figure 6:**
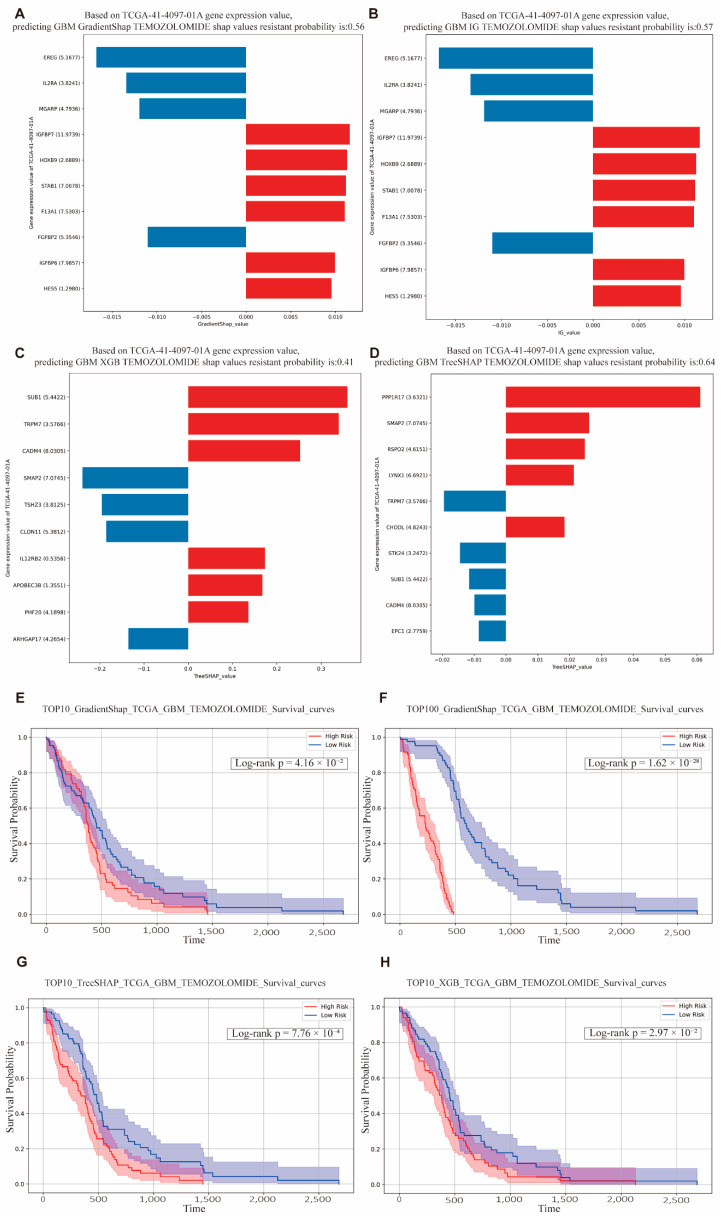
Prediction of temozolomide resistance and survival prognosis in GBM patients. (**A**–**D**) display the distributions of SHAP values from four models predicting temozolomide resistance in GBM patients. The x axis shows SHAP values, and the y axis lists genes together with their expression levels. Blue: the gene and its expression contribute negatively to the prediction of resistance; bar length indicates the magnitude of the negative contribution. Red: the gene and its expression contribute positively to the prediction of resistance; bar length indicates the magnitude of the positive contribution. (**A**) The probability of predicting patient resistance based on the GradientSHAP algorithm was 0.56, and the relevant gene expression analysis. (**B**) The probability of predicting patient resistance based on the IG algorithm was 0.57, and the relevant gene expression analysis. (**C**) The probability of predicting patient resistance based on the XGB algorithm was 0.41, and the relevant gene expression analysis. (**D**) The probability of predicting patient resistance based on the XGB algorithm was 0.64, and the relevant gene expression analysis. (**E**) Using the GradientSHAP interpretable method, the top 10 genes were selected as a gene set for survival analysis in GBM patients, with high-risk patients having significantly shorter survival times than low-risk patients. The genes selected by IG were consistent. (**F**) The top 100 genes were used as a gene set for survival analysis in GBM patients, and the survival time of high-risk patients was significantly shorter than that of low-risk patients, with a *p* value of 1.6159 × 10^−28^. (**G**) Based on the TreeSHAP interpretable method, the top 10 genes were used as a gene set for survival analysis in GBM patients, the survival rate of high-risk patients was lower, with *p* far less than 0.05. (**H**) Using the XGB interpretable method to select the top10 genes as a gene set for survival analysis in GBM patients, the survival time of high-risk patients was shorter, with *p* far less than 0.05.

Based on the aforementioned model, we selected the five GBM patients with the longest survival times and the patient with the shortest survival time for detailed predictive drug response analysis. Among the patients with the shortest survival durations, the VAE_LD model combined with IG/GradientSHAP predicted drug resistance probabilities of 0.56 and 0.57, respectively, indicating a high degree of consistency (Figure 6A,B). Importantly, the key contributing genes were also consistent across these cases, with *IGFBP7* emerging as the most influential gene in resistance prediction. Previous studies have demonstrated that *IGFBP7* is significantly associated with poor prognosis in GBM and promotes tumor angiogenesis, thereby contributing to drug resistance [37,38].

In comparison, machine learning models XGB and TreeSHAP predicted drug resistance probabilities of 0.41 and 0.64, respectively (Figure 6C,D). Interestingly, although *SUB1* was not the most highly expressed gene in the XGB model, it was identified as a key contributor to resistance. Prior studies have shown that *SUB1* is upregulated in GBM and enhances tumor cell proliferation and migration. According to TreeSHAP, the gene *PPP1R17*, despite its relatively low expression, accounted for a substantial proportion of resistance contribution. Research by Tokizane et al. found that neurons with high *Ppp1r17* expression are associated with aging phenotypes, and inhibition of *Ppp1r17* can alleviate neurological symptoms and extend lifespan in mouse models [39]. This suggests a potential mechanism by which GBM may promote drug resistance through aging-related pathways involving *PPP1R17*. Predicted drug resistance probabilities for other patients with short survival times also ranged between 0.5 and 0.9, reflecting substantial inter-individual variability.

Among the five patients with the longest survival durations, the VAE_LD and IG/GradientSHAP model predicted drug sensitivity probabilities of around 0.45 in some patients (Supplementary Figure S11), while others showed predicted probabilities as high as 1.0 (Supplementary Figure S11), again indicating pronounced individual variation. In patients with a predicted sensitivity probability of 1.0, the most important genes identified were *NOTCH3*, *SOD3*, and *NR2E1*.

*NOTCH3* is mainly involved in angiogenesis and is expressed in brain tissues. While it functions as an oncogene in many cancer types, some studies have reported a tumor-suppressive role. In the context of GBM, *NOTCH3* expression has been detected in some drug-resistant strains, while absent in others. These findings suggest that high *NOTCH3* expression may sensitize GBM cells to chemotherapy, although the role remains context-dependent [40,41]. *SOD3* and *NR2E1* are known tumor suppressor genes, and both have been previously implicated in GBM, supporting their role in promoting treatment sensitivity.

The interpretable algorithms of the two machine learning models predicted sensitivity probabilities of 0.61 (TreeSHAP) and 0.69 (XGB) (Supplementary Figure S11), respectively. The associated gene contributions also varied significantly. In XGB, *CIAPIN1* was found to be highly expressed and identified as a major regulator of apoptosis. Its elevated expression correlated positively with treatment sensitivity. Conversely, the TreeSHAP model considered *CIAPIN1* to be a non-contributory gene and instead highlighted *APOBEC3D*, which is known to drive tumor resistance [42]. This discrepancy persisted across repeated evaluations and may reflect model-specific differences in feature attribution. Additional analyses of drug sensitivity and resistance across other tumor types are provided in the Supplementary Materials. (See Supplementary Figure S11 for details).

In the TCGA-based survival analysis, the top 10 and top 100 genes identified by the models were used to construct gene sets, where high expression levels indicated high risk, and low expression levels indicated low risk. The results showed that, regardless of the algorithm applied, patients in the high-risk group exhibited significantly lower overall survival compared to those in the low-risk group, with *p* values well below 0.05 (Figure 6E). Moreover, the top 100 gene (T100) sets yielded even more statistically significant survival differences (Figure 6F).

These findings suggest that the gene sets identified by the VAE_LD-based model and its associated interpretability algorithms (IG/GradientSHAP) can serve as robust prognostic biomarkers for predicting poor clinical outcomes.

Similarly, the top 10 and top 100 gene sets derived from the TreeSHAP and XGBoost (XGB) algorithms also showed significant differences in survival between risk groups (*p* < 0.05) (Figure 6G,H). While gene sets containing 100 genes provide increased statistical power due to the larger number of included features, their clinical utility is limited by the practical challenges of obtaining and analyzing large gene expression panels in routine clinical settings. In contrast, the top 10 gene sets offer a more feasible and clinically applicable solution, while still maintaining strong statistical significance (Table 4). These smaller, high-impact gene panels hold promise for implementation in precision oncology workflows. Prognostic analyses for additional cancer types are provided in the Supplementary Materials. (See Supplementary Figure S12 for details).

## 4. Discussion

This study integrates large scale transcriptomic modeling with interpretable artificial intelligence to tackle the heterogeneity of tumor drug resistance in both preclinical and clinical settings. Five distinct models (VAE_LL, VAE_LS, RF, XGB, and VAE_LD) were retrained on the GDSC cell line dataset containing 72 chemotherapeutic agents, enabling the capture of broad resistance patterns across diverse tumor contexts. The four best performing models, selected according to predictive metrics, were then validated in five TCGA cancer cohorts with a total of 1836 patients. For each cancer type, response to nine clinically relevant first line drugs was modeled, resulting in 180 prediction tasks that span all drug and cancer combinations. This multi-level experimental design provides a pragmatic bridge from in vitro drug sensitivity profiling to clinically grounded biomarker discovery and risk stratification, with resulting clinical inferences considered hypothesis-generating rather than confirmatory.

VAE_LD, which applies a knowledge distillation strategy, achieved the highest accuracy, F1 score, and AUC on the GDSC training set (average AUC 0.81 and F1 score 0.92). These results suggest potential generalizability within preclinical settings, with clinical generalizability contingent on validation in treatment-annotated cohorts. By adapting the Deep learning transferred framework to fit bulk RNA sequencing, we enabled efficient transfer of models trained on cell lines to patient cohorts. This methodological adaptation underscores the flexibility of the framework and its potential translational relevance; however, real-world clinical utility remains to be established.

To ensure biological interpretability, we applied Integrated Gradients, GradientSHAP, and TreeSHAP to interrogate feature importance and reveal underlying mechanisms. In glioblastoma treated with Temozolomide, the models prioritized *OPALIN*, *LTF*, *IL2RA*, and *SLC17A7* as candidate resistance related genes. Although these genes have known roles in tumor biology, their specific contributions to Temozolomide resistance are not well defined, suggesting the need for further experimental verification [43] and indicating that these signals should be interpreted as hypothesis-generating. Gene interaction network analysis provided another layer of support. For instance, interactions between *LTF* and *ADAMTS16* in glioblastoma indicate a possible role in epithelial to mesenchymal transition. Functional enrichment pointed to granulocyte recruitment, angiogenesis, and cancer stemness, reinforcing the biological plausibility of the predicted biomarkers.

Beyond correlation, several features support a causal role for the highlighted markers in context. In LUAD, *TFF1* and *B3GNT6* converge on epithelial differentiation and mucin-linked receptor trafficking, a biology that can modulate EGFR-TKI dependence and thereby rationalize resistance to Gefitinib. In GBM, *LTF* and *IL2RA* align with immune-evasive and angiogenic programs that are mechanistically plausible mediators of Temozolomide response, whereas *SLC17A7* tracks with neuronal differentiation and has been reported to oppose proliferative signaling, consistent with a sensitivity-associated role. *OPALIN* behaves as a lineage marker, suggesting that lineage state rather than direct effector function may underlie its association. These convergences across pathway level, lineage context, and established drivers argue for biological plausibility. However, patient-level signals remain hypothesis-generating and require orthogonal validation in treatment-annotated cohorts and experimental perturbations to establish causality.

VAE_LS consistently underperformed relative to VAE_LL, VAE_LD, RF, and XGB. Possible reasons include limited sample size, uneven RNA sequencing quality, and mismatch between model complexity and dataset scale. Validation in larger, higher quality datasets will be necessary to refine architecture choices and confirm these observations [44,45]. Classical machine learning models such as RF and XGB delivered stable performance and, when coupled with TreeSHAP, successfully identified biologically meaningful genes including *TRPM7*, *CHODL*, and *SMAP2*. These genes were enriched in pathways related to angiogenesis, epithelial to mesenchymal transition, and immune regulation. This finding underscores the value of ensemble methods for mechanistic discovery, even if their predictive metrics are slightly lower than those of VAE_LD [46]. However, such models may struggle in complex clinical contexts because they rely on one dimensional gene features and do not fully capture inter patient heterogeneity. In contrast, deep learning frameworks, especially VAE based architectures, excel at learning non-linear representations and integrating modular biological signals, which makes them better suited for multiomics integration and interpretation.

Moreover, there are several shared limitations that warrant further clarification. First, the study depends on GDSC derived cell line data that mainly includes traditional chemotherapeutic agents and does not incorporate modern immunotherapies. Second, TCGA cohorts lack comprehensive treatment exposure and response annotations, and often exhibit variable sequencing quality, which means our external analyses are correlative and should be viewed as hypothesis-generating rather than confirmatory [47]. GDSC offers high-throughput pharmacogenomic measurements in immortalized cell lines under controlled conditions, which facilitates comparative modeling but does not recapitulate stromal interactions, immune contexture, or pharmacokinetics in patients; plate conditions, assay protocols, and release-to-release differences can introduce technical heterogeneity that may affect generalizability. TCGA provides large, multi-center bulk transcriptomes with survival follow-up but lacks standardized, patient-level treatment exposure and response endpoints, and several clinical fields are incomplete or inconsistently annotated across disease programs. Variation in sequencing centers and preprocessing, together with differences in tumor purity and stromal admixture, can further modulate transcriptomic signals and complicate cross-tumor comparisons. These factors mean that the patient-level associations reported here are exploratory and hypothesis-generating rather than confirmatory. Where possible, harmonized preprocessing and prespecified correction procedures were applied to reduce technical variation, and claims have been limited to what is supported by the available data. Prospective evaluation in independent, treatment-annotated cohorts, together with functional validation in patient-derived systems, will be essential to determine whether the prioritized biomarkers and pathways add value beyond established clinical factors.

Future work should prioritize multicenter clinical datasets with detailed treatment metadata, develop AI frameworks that extract robust biological signals from small, high-quality real-world cohorts, and integrate additional molecular layers, including epigenomic, proteomic, and mutational profiles, to enhance robustness and clinical applicability [48]. Beyond expression profiles, a multi-omics view is likely to strengthen both discrimination and mechanistic plausibility. Somatic drivers and copy number states provide complementary constraints on pathway dependence and can be incorporated as inputs to shared latent representations; for example, modeling interactions between *EGFR* and *ALK* alterations in LUAD, or conditioning on *IDH1* status and *MGMT* promoter methylation in GBM when evaluating temozolomide response. Epigenetic context, including DNA methylation programs that modulate DNA-repair capacity and immune trafficking, can help distinguish lineage or microenvironmental influences from causal resistance mechanisms. Proteomic and phosphoproteomic measurements capture pathway activity not always apparent at the mRNA level and are particularly relevant for signaling nodes such as the VEGF–VEGFR axis, where protein abundance and phosphorylation states may mediate anti-angiogenic response more directly than VEGFA transcription. Methodologically, joint modeling can be framed with shared and private latent factors that fuse modalities while preserving modality-specific signal, with explicit handling of missing blocks and harmonized normalization to mitigate platform heterogeneity. Because multi-omics integration often reduces the number of complete cases, careful attention to imputation, sensitivity analyses, and external validation in treatment-annotated cohorts will be essential. Within this framework, the transcriptomic biomarkers highlighted here should be viewed as hypothesis-generating anchors that motivate multi-omics follow-up rather than standalone determinants of resistance.

The combination of VAE_LD and SHAP based interpretation provides a transparent analytic platform for hypothesis generation in modeling drug resistance mechanisms. This framework supports the prioritization of candidate biomarkers, reconstruction of resistance related signaling networks, and integration with survival modeling, while its translational utility will require confirmation in independent, treatment-annotated cohorts. Although multiple novel targets were identified across cancer types, experimental validation and evaluation in clinical cohorts with documented regimens and response endpoints is still required to establish their causal involvement in resistance.

Moving forward, the field should gradually transition from reliance on public resources like GDSC and TCGA to multi center, clinically annotated datasets that include documented therapies and on-treatment response measures. A systematic comparison of machine learning and deep learning models will be essential to establish standardized pipelines that meet the demands of precision medicine [49]. In conclusion, the VAE_LD centered framework, trained on 72 drugs and validated through 180 drug and cancer prediction tasks, provides hypothesis-generating estimates of resistance risk using bulk RNA sequencing data and reveals putative molecular mechanisms and prognostic associations. With continued optimization in real world clinical cohorts and expansion to multi-omics integration, this approach holds strong promise for biomarker discovery, patient stratification, and clinical decision support in individualized cancer therapy.

## 5. Conclusions

In conclusion, an interpretable transfer-learning framework centered on a residual variational autoencoder was trained on GDSC (72 agents) and explored across five TCGA cancer types (*n* = 1836), yielding strong in vitro discrimination and consistent cross-tumor resistance signals across 180 tumor–drug tasks. The approach prioritizes biologically plausible candidate biomarkers and pathways and provides a transparent link from model attributions to mechanisms. As exemplars rather than confirmatory markers, *TFF1* and *B3GNT6* were repeatedly associated with Gefitinib resistance in LUAD, while *OPALIN, LTF*, *IL2RA*, and *SLC17A7* were implicated in Temozolomide response in GBM, aligning with processes such as epithelial differentiation and angiogenesis. Because TCGA lacks treatment-response labels, all patient-level inferences are exploratory and hypothesis-generating. Practical translation will require validation in independent, treatment-annotated cohorts with standardized response endpoints and time-to-event measures, orthogonal confirmation in patient-derived models and targeted perturbation assays, and assay/reporting standardization for compact panels together with decision analytic evaluation of net clinical benefit. To facilitate reproduction and extension, all code, containers, configuration files, exact train–validation splits, and the end-to-end workflow (BPMN plus pseudocode) are publicly released under a permanent identifier. Subject to these validations and with expanded multi-omics integration, the framework has clear potential to support biomarker discovery and patient stratification in precision oncology.

Future work guidelines: Translation to practice requires validation in multi-institutional, treatment annotated cohorts with harmonized covariates and standardized endpoints, including objective response and time to event outcomes. Performance should be reported with discrimination and calibration together with clinical utility quantified by decision curve analysis under prespecified, locked thresholds. Robustness must be demonstrated across centers, platforms, sampling procedures, and patient subgroups, with sensitivity analyses for distribution shift, missingness, tumor purity, and other confounders, and with explicit procedures for detecting samples outside the training distribution. Interpretability should be reproducible and decision relevant by fixing attribution pipelines a priori, testing stability across resamples, and showing pathway level coherence rather than isolated features. Biological credibility should be examined through gain or loss of function perturbations in isogenic lines and patient derived organoids, dose–response assays, rescue experiments, and single cell or spatial profiling before and after drug exposure. Multi-omics integration that combines transcriptomics with copy number, methylation, chromatin accessibility, proteomics, and pathology should be assessed for incremental clinical utility under identical validation plans. Prospective evaluation should include a registry-based implementation study followed by a pragmatic, biomarker informed trial comparing a prespecified strategy with usual care. Reproducibility and governance should be ensured with versioned containers, model cards, data dictionaries, audit trails, fairness analyses, and documented privacy safeguards.

## Figures and Tables

**Table 1:** Predictive performance of different models based on 72 drugs.

Model	AUC	MCC	F1 Score	Recall	Accuracy	Precision
VAE_LD	0.807	0.366	0.894	0.914	0.860	0.881
RF	0.801	0.355	0.893	0.913	0.855	0.877
VAE_LS	0.650	0.183	0.862	0.889	0.811	0.838
VAE_LL	0.784	0.343	0.891	0.911	0.848	0.873
XGB	0.791	0.336	0.895	0.924	0.860	0.871

**Table 2:** Predictive performance of different models based on 9 drugs.

Model	AUC	MCC	F1 Score	Recall	Accuracy	Precision
VAE_LD	0.814	0.381	0.852	0.848	0.810	0.889
RF	0.806	0.372	0.832	0.834	0.808	0.833
VAE_LS	0.652	0.136	0.795	0.824	0.776	0.773
VAE_LL	0.783	0.322	0.828	0.843	0.803	0.815
XGB	0.804	0.350	0.834	0.841	0.817	0.828

**Table 3:** Ranking of relevant genes in tumor resistance/sensitivity.

Feature_Name	Sensitive_Rank	Resistant_Rank
**GradientSHAP**		
*OPALIN*	3	2
*LTF*	9	1
*IL2RA*	5	8
**IG**		
*OPALIN*	3	2
*LTF*	9	1
*IL2RA*	5	8

**Table 4:** Based on different models and interpretable methods, the top 10 genes most important for predicting the prognosis of GBM patients were selected.

GradientSHAP	IG	RF	XGB	GradientSHAP∩IG	Rank
*SAA1*	*SAA1*	*TRPM7*	*SUB1*	*ADAMDEC1*	1
*LTF*	*LTF*	*CHODL*	*TRPM7*	*ANKRD7*	2
*PPBP*	*PPBP*	*SMAP2*	*CLDN11*	*AQP5*	3
*GSTM1*	*GSTM1*	*APOBEC3D*	*ZFPM2*	*BIRC7*	4
*MOBP*	*MOBP*	*LYNX1*	*CHODL*	*CACNG3*	5
*VGF*	*VGF*	*CIAPIN1*	*CIAPIN1*	*CAPS*	6
*PLA2G2A*	*PLA2G2A*	*CHRDL1*	*IL12RB2*	*CARNS1*	7
*CXCL14*	*CXCL14*	*SUB1*	*SMAP2*	*CCK*	8
*ADAMDEC1*	*ADAMDEC1*	*PPP1R17*	*CADM4*	*CD163*	9
*NNAT*	*NNAT*	*PBX2*	*BEX1*	*CD24*	10

## Data Availability

All of our data were obtained from publicly available databases and can be accessed through the corresponding websites.

## Supplementary Materials

The supplementary materials supporting the findings of this study are publicly available in the Zenodo repository and can be accessed via https://doi.org/10.5281/zenodo.17090854. The corresponding DOI is https://zenodo.org/records/17090854 (15 June 2025).
